# Impact of antibiotic prophylaxis on gut microbiota in colorectal surgery: insights from an Eastern European stewardship study

**DOI:** 10.3389/fcimb.2024.1468645

**Published:** 2025-01-13

**Authors:** Irina Cezara Văcărean-Trandafir, Roxana-Maria Amărandi, Iuliu Cristian Ivanov, Loredana Mihaiela Dragoș, Mihaela Mențel, Ştefan Iacob, Ana-Maria Muşină, Elena-Roxana Bărgăoanu, Cristian Ene Roată, Ștefan Morărașu, Valeri Țuțuianu, Marcel Ciobanu, Mihail-Gabriel Dimofte

**Affiliations:** ^1^ TRANSCEND Research Centre, Regional Institute of Oncology, Iasi, Romania; ^2^ Second Surgical Oncology Department, Regional Institute of Oncology, Iasi, Romania; ^3^ Surgery Department, “Grigore T. Popa” University of Medicine and Pharmacy, Iasi, Romania; ^4^ Scientific Laboratory of Cancer Biology, Institute of Oncology, Chișinău, Moldova; ^5^ Surgical Oncology Department, Proctology, Institute of Oncology, Chișinău, Moldova

**Keywords:** gut microbiota, colorectal cancer, antibiotic therapy, 16S rRNA NGS, ASP, AMS, LMICs

## Abstract

**Introduction:**

Antibiotic overuse is driving a global rise in antibiotic resistance, highlighting the need for robust antimicrobial stewardship (AMS) initiatives to improve prescription practices. While antimicrobials are essential for treating sepsis and preventing surgical site infections (SSIs), they can inadvertently disrupt the gut microbiota, leading to postoperative complications. Treatment methods vary widely across nations due to differences in drug choice, dosage, and therapy duration, affecting antibiotic resistance rates, which can reach up to 51% in some countries. In Romania and the Republic of Moldova, healthcare practices for surgical antibiotic prophylaxis differ significantly despite similarities in genetics, culture, and diet. Romania's stricter healthcare regulations result in more standardized antibiotic protocols, whereas Moldova's limited healthcare funding leads to less consistent practices and greater variability in treatment outcomes.

**Methods:**

This study presents the results of a prospective cross-border investigation involving 86 colorectal cancer patients from major oncological hospitals in Romania and Moldova. We analyzed fecal samples collected from patients before and 7 days post-antibiotic treatment, focusing on the V3–V4 region of the 16S rRNA gene.

**Results:**

Our findings indicate that inconsistent antibiotic prophylaxis policies—varying in type, dosage, or therapy duration—significantly impacted the gut microbiota and led to more frequent dysbiosis compared to stricter prophylactic antibiotic practices (single dose, single product, limited time).

**Discussion:**

We emphasize the need for standardized antibiotic prophylaxis protocols to minimize dysbiosis and its associated risks, promoting more effective antimicrobial use, particularly in low- and middle-income countries (LMICs).

## 1 Introduction

It is estimated that 310 million major surgeries are being performed worldwide each year (Dobson, 2020), with every procedure carrying a significant risk of surgical site infections (SSIs). This risk dramatically increases if no antibiotic prophylaxis is administered (World Health Organization, 2018; Cooper et al., 2020; Dona et al., 2020; Takayama et al., 2019; Ariyo et al., 2019). Prior to the use of prophylactic antibiotics, SSIs were the major cause of nosocomial infections, with substantial fatality rates. Antibiotic prophylaxis is one of the primary strategies for lowering SSI rates by reducing bacterial burden, and antibiotics are administered intravenously or orally, often in accordance with specific guidelines (Gaynes et al., 2001; Smyth and Emmerson, 2000; Humphreys, 2009). Despite its effectiveness in reducing SSIs, antibiotic prophylaxis regimens vary considerably; some institutions opt for a perioperative single dose of a second-generation cephalosporin, while others allow surgical teams to select the antibiotic regimen without imposing a standardized protocol (Badia et al., 2017; Allegranzi et al., 2018; Meara et al., 2015; Anderson et al., 2008). Thus, it is not uncommon for antibiotic prophylaxis to be performed on an *ad hoc* basis, especially in countries that have not yet achieved harmonization of healthcare practices.

There is limited understanding of the immediate and short-term effects of antibiotics on complex bacterial populations, with each antibiotic prescription requiring a careful balance of advantages and risks to both the patient and the community. The stress we put on the intestinal microbiota, primarily through antibiotic overuse, can lead to severe diseases such as *Clostridioides difficile* infection, adversely affecting patients’ gut microbiota. The magnitude of this impact could encourage adjustments in antibiotic prescribing practices and patient health behaviors. With an extensive variety of antibiotics available, several broad-spectrum antimicrobial agents are frequently used on an empirical basis, especially in hospitals. This practice is considered an “efficient” approach for treating infections, as it ensures comprehensive coverage against a broad array of bacteria. However, this hasty practice has led to a substantial increase in antibiotic-resistant diseases. Antibiotics have been shown to destroy commensal and sensitive bacteria while allowing resistant species to proliferate and eventually colonize the mucosa (Langdon et al., 2016; Patrick and Hutchinson, 2009). Furthermore, these resistant species can be passed on to other patients, leading to nosocomial infections. As a result, antibiotic abuse prevention initiatives have recently become increasingly popular, raising awareness about the significant role of antibiotics in bacterial dysbiosis (Ramirez et al., 2020).

As *Clostridioides difficile* infections have emerged as common and serious complications among hospital patients, Western European countries have particularly begun advocating for more cautious antibiotic administration. Tang et al. published a meta-analysis on *Clostridioides difficile* infections that concluded that, in addition to prolonged hospitalization and proton pump inhibitors, antibiotic usage was the greatest risk factor for *Clostridioides difficile* infection incidence (Tang et al., 2016; Arriola et al., 2016; Baur et al., 2017). *Clostridioides difficile* infections have increased two to fourfold in the last decade, mirroring a significant increase in broad-spectrum antibiotic misuse (Honda et al., 2017; Ho et al., 2020). Antibiotic overuse not only increases the probability of *Clostridioides difficile* infection but also disrupts the interactions between the gut microbiota, immune system, and hormonal system. Many studies have linked this imbalance to major disorders, such as inflammatory bowel disease (IBD) (Deshpande et al., 2013; Issa et al., 2007; Rodemann et al., 2007; Ananthakrishnan et al., 2009; Goodhand et al., 2011) and depression (Evrensel and Ceylan, 2015; Makris et al., 2021; Zalar et al., 2018). As a result, the World Health Organization (WHO) launched a global campaign against antimicrobial resistance. Reports indicate that due to the wide variety of available antibiotics, treatment protocols vary substantially between nations, not only in terms of the administered medication but also in terms of the dosage and duration of treatment. This variation contributes to the significant discrepancies in antibiotic resistance rates between countries, which range from 0% to 51% (World Health Organization, 2023).

Antibiotic consumption surveillance is an essential aspect of antimicrobial stewardship programs (ASPs), detailing the frequency and trends of use within and across medical centers. These statistics can then indicate instances of inappropriate antibiotic use and highlight the need for further examination or remediation (Charani et al., 2019; Cox et al., 2017; Arulappen et al., 2020; Allegranzi et al., 2016). A comprehensive review by Cooper et al. on antibiotic prophylaxis for the prevention of surgical site infections in low- and middle-income countries (LIMCs), supported by numerous studies (GlobalSurg, 2018; Allegranzi et al., 2011; Branch-Elliman et al., 2019; Butt et al., 2019; Founou et al., 2017), revealed that guidelines on the use of surgical antibiotic prophylaxis (SAP) are not always followed. As such, many surgeons in developing countries continue to administer antibiotics postoperatively (Cooper et al., 2020). According to general guidelines, primary surgical antimicrobial prophylaxis should be administered one hour before the operation and augmented by further doses if the surgery lasts more than two antibiotic half-lives. Antibiotic administration should not be prolonged for more than 24 hours following surgery, and a single preoperative dose is generally sufficient (Bratzler et al., 2013). Cefuroxime, a second-generation cephalosporin, is the antibiotic of choice for a variety of applications, from surgical prophylaxis and complex fractures to simple abrasion injuries. Furthermore, it is among the most stable β-lactam antibiotics used to lower the risk of postoperative SSIs, sepsis, or abscesses, and is also cost effective, having proven its efficacy in numerous clinical trials (Bratzler et al., 2013; Sastry et al., 2021). Notably, several studies on antibiotic prophylaxis with cefuroxime, in combination with mechanical bowel preparation (MBP) for colorectal surgeries have reported a reduction in SSIs and bacterial burden (Rollins et al., 2019; Saha et al., 2014; Toh et al., 2018; Poggio, 2013). However, further high-quality research on changes in the gut microbiota is necessary to encourage more individualized antibiotic treatment schemes in conjunction with MBP aimed at minimizing surgical site infections (Grewal et al., 2021).

Colorectal cancer (CRC) is the third most frequently diagnosed cancer worldwide, with recent data indicating the highest mortality rates in Eastern Europe. Considering the existing and prospective burden of CRC in 185 countries, data from GLOBOCAN indicate that the number of CRC cases and related mortality will double by 2040. The geographical and temporal distribution of CRC provides insights into the prevalence of risk factors and advances in cancer control techniques (Morgan et al., 2023; O'Sullivan et al., 2022; Arnold et al., 2017). Despite developments in surgical methods and antiseptic measures over the last few decades, colorectal cancer surgery is still associated with high infection morbidity (Artinyan et al., 2015; Nelson et al., 2014). Recent studies in the field of colorectal surgery have shown that the microbiome significantly influences postoperative outcomes, particularly the occurrence of SSIs and anastomotic leakage (Bartolini et al., 2020; Krezalek and Alverdy, 2023; Guyton and Alverdy, 2017). The current strategy for managing high bacterial burdens before surgery involves maximizing decontamination through antibiotic prophylaxis alone or in combination with MBP. Unfortunately, this decontamination affects both beneficial and potentially harmful bacteria. Currently, there is a considerable literature gap regarding the detailed description of intestinal microbiota modifications in patients following colon surgery, particularly with regard to state-of-the-art next-generation sequencing (NGS) assays. With advances in NGS technology and metagenomics, coupled with an increased interest in understanding the physiology of the gut microbiota and its disruption under antibiotic stress, a focused research pool has emerged since 2021, linking gut microbiota dynamics to CRC (Dulal and Keku, 2014; Keku et al., 2015; An et al., 2021; Abbas et al., 2022; Hunter, 2023; Pandey et al., 2023).

Therefore, we present the results of a cross-border study conducted between two oncological hospitals in Romania and the Republic of Moldova to assess the impact of different antibiotic prophylaxis protocols on the intestinal microbiota of two similar patient populations who share nearly identical culinary practices and environmental conditions. At the Regional Institute of Oncology Iasi (IRO) in Romania, a strict protocol was employed consisting of a single intravenous dose of second-generation cephalosporin (cefuroxime) plus metronidazole. In contrast, the National Institute of Oncology from Moldova (NIO) in Chisinau allowed for a more permissive approach consisting of multiple drug combinations, including first- and third-generation cephalosporins (cefazolin, cefotaxime, ceftriaxone and cefoperazone in combination with sulbactam), and longer periods of antibiotic use for nonprophylactic regimens. By collecting paired samples from the same patients over the same time span, we aimed to assess how different antibiotic therapy protocols affect the gut microbiota. Additionally, in the same cohort of surgical oncological patients, we evaluated the ability of the intestinal microbiota to short-term self-regulate by examining and comparing its structure 7 days after antibiotic modifications. We characterized the intestinal microbiota of a total of 400 surgical oncological patients (200 from each center) to determine whether the overall microbial community composition differed between these groups and within each group, with partial results from one center being previously published (Văcărean-Trandafir et al., 2023). Here, we present the findings on 86 selected colorectal cancer patients from each group and discuss how different antibiotic prophylaxis protocols impact the intestinal microbiota 7 days after the end of antibiotic treatment.

## 2 Materials and methods

### 2.1 Study design and sample collection

#### 2.1.1 Location

This cross-border study was carried out at two major oncology hospitals: in the eastern part of Romania at the Regional Institute of Oncology in Iasi - hereafter referred to as IRO and in the Republic of Moldova at the National Institute of Oncology in Chisinau, hereafter referred to as NIO. Each serves a population of approximately 4 million patients. Both hospitals have an occupancy capacity of more than 330 beds and provide a range of services, including intensive care, general surgery (thoracic, plastic, gynecologic, etc.), medical oncology, hematology and radiotherapy. Adjacent research laboratories are present in both institutes and at IRO, there is also a dedicated research center, TRANSCEND, where the molecular analyses were performed. Ethical board approval for the study protocol and sample size was obtained from the IRO/NIO Clinical Research Ethics Committee prior to the initiation of this study.

#### 2.1.2 Participants and sample collection

A total of 400 surgical oncological patients were enrolled in the study, with 200 from each oncology hospital (IRO Romania and NIO Moldova), totaling 800 paired samples. Patients were hospitalized for noninfectious diseases and had not received any antibiotic treatment three months prior to admission. Sampling occurred consecutively from April 2021 to January 2023, and all participants qualified for prophylactic antibiotic medication. The inclusion criteria were adults undergoing any type of surgery requiring antibiotic prophylaxis, with or without MBP, and a 21-day break from any neoadjuvant treatment, such as chemotherapy or radiation, if applicable. Participants were required to provide written informed consent for fecal sampling and processing. The exclusion criteria included patients who had received systemic or oral antibiotic treatment in the previous 30 days (for infectious diseases such as bacterial urinary tract infections), had a history of MBP in the previous month, had undergone ileostomy at the time of admission, experienced late resumption of intestinal transit (more than ten days post-surgery), had occlusive syndromes, or required surgery without antibiotic prophylaxis (such as breast surgery or plastic surgery). At IRO, all patients received a standard prophylactic antibiotic regimen preoperatively, adhering to clinical practice guidelines for antimicrobial prophylaxis during surgery (Bratzler et al., 2013): a single dose of 1.5g cefuroxime administered intravenously before the incision, with up to two doses within 24 hours, depending on the length of the surgery, plus a single dose of 2g metronidazole. At NIO, patients were treated with various cephalosporins, including cefazolin (first generation), cefotaxime, ceftriaxone and cefoperazone in combination with sulbactam (third generation cephalosporins), each at a dosage of 1g/day, for various durations, with or without two doses of 0.5g metronidazole/day, and for various durations. For the current analysis, from this cohort of 400 patients, we selected 86 patients who underwent colorectal surgery for oncological reasons (50 cases from the NIO cohort and 36 from the IRO cohort, comprising 172 paired samples).

For the molecular analysis of the gut microbiota, we collected paired stool samples from each patient: a preoperative/pretreatment (M) sample and a postoperative (T) sample taken 7 days after the end of antibiotic treatment. If MBP was performed in addition to systemic antibiotic prophylaxis, the M sample was collected prior to MBP. Each sample was collected by rectal examination as previously described (Văcărean-Trandafir et al., 2023), in accordance with methods used in other comparative studies (Schlebusch et al., 2022; Short et al., 2021; Besasie et al., 2019; Reyman et al., 2019; Radhakrishnan et al., 2023; Budding et al., 2014). Rectal examination was chosen as the sample collection method rather than fecal deposition because it minimizes variability in sample collection and is a routine part of the patient’s preoperative care, thereby preventing the patient from being subjected to additional procedures. After collection, the samples were immediately refrigerated at 4°C if they were to be analyzed shortly thereafter or stored frozen at -20°C until DNA isolation could be performed.

#### 2.1.3 Demographic information assessment

The data collected included patient demographic information; tobacco usage; diagnosis; type of surgery; MBP status; results of GDH + Tox A + Tox B analysis for *Clostridioides difficile*; multidrug-resistant organisms; extended-spectrum beta-lactamase (ESBL) testing; antibiotic prophylaxis, total number and types of prophylactic doses; and duration of antibiotic treatment. This information was collected from the patients’ recorded data sheets in accordance with written consent forms.

### 2.2 Sample processing and sequencing

#### 2.2.1 DNA isolation

The DNA isolation procedure was performed on batches of 12-16 samples to prevent cross-contamination using the NucleoSpin^®^ Soil Kit (Macherey-Nagel, Düren, Germany), as previously described (Văcărean-Trandafir et al., 2023). The samples were vigorously vortexed with lysis buffer and proteinase K and then incubated overnight at 37°C. Both enzymatic and mechanical lysis methods were combined for the optimal breakdown of bacteria. Finally, the gDNA was eluted in 30 μL of SE buffer and stored at -20°C. No-template controls were also included in each sequencing run for quality assurance.

The concentration and purity of the isolated DNA were measured spectrophotometrically using a NanoDrop device (Thermo Fisher Scientific, Massachusetts, USA). The integrity and size of the DNA were assessed qualitatively by gel electrophoresis on a 2% agarose gel (w/v) stained with 0.5 μg/ml ethidium bromide and run in 1x TAE buffer at a constant voltage of 180 V.

#### 2.2.2 16S rRNA gene amplification and sequencing

The 16S Metagenomic Sequencing Library Preparation protocol from Illumina was used for bacterial composition characterization (Illumina, 2013). DNA was PCR amplified as previously described (Văcărean-Trandafir et al., 2023) using primers specific for the V3-V4 region to create ~460 bp amplicons. PCR products were purified with magnetic beads on a BIOMEK^®^ FXP workstation (Beckman Coulter, Brea, US). Afterwards, Nextera XT indexes and sequencing adapters were added to the PCR mixture as previously indicated (Văcărean-Trandafir et al., 2023). After purification and quantification, libraries were pooled, denatured to a final concentration of 12 pM, mixed with 20% Phix control (Illumina, San Diego, USA), and sequenced on an Illumina MiSeq platform using a 600-cycle (2 × 301 bp) MiSeq reagent kit for paired-end sequencing.

#### 2.2.3 Quality control

All samples analyzed in this study underwent DNA extraction and sequencing in the same laboratory, and laboratory personnel were blinded to the case status. A total of five sequencing runs (96 samples each) were performed: four for the IRO samples and one for the NIO samples. Both positive control samples and negative controls were included across all sequencing batches. Fifteen DNA negative extraction control blanks consisting of 10 mM Tris-HCl (pH 8) were used throughout the process to identify potential contaminants.

An artificial (mock) community was established using in-house strains reflecting a variety of bacterial taxa, including gram-positive bacteria (*Enterococcus faecalis, Staphylococcus aureus, and Staphylococcus epidermidis*) and gram-negative bacteria (*Escherichia coli, Klebsiella pneumoniae, Serratia marcescens*). This mock community served as a control to verify the accuracy of the DNA extraction method. The mock community was prepared by combining equal volumes of cell suspensions from each of the bacterial strains (between 10^7^ and 10^8^ cells), thereby ensuring an equal representation of each strain in the mixture, as previously stated (Văcărean-Trandafir et al., 2023). We included a total of twelve positive controls randomly distributed in the sequencing runs.

### 2.3 Bioinformatic and statistical analysis

#### 2.3.1 Sequence processing and taxonomic profiling

Demultiplexed sequences were processed using the DADA2 pipeline (Callahan et al., 2016) in R (version 4.1.2), with minor modifications, as previously described (Văcărean-Trandafir et al., 2023). Briefly, forward and reverse reads were stripped of primers and trimmed to 255 and 215 bases, respectively. Sequences containing ambiguous bases were discarded. Pairs were merged, chimeras were detected and removed, and merged sequences with fewer than 350 bases were excluded from further analyses. Taxonomy was assigned using the RDP Naive Bayesian classifier implemented in dada2, employing either the SILVA database (release 138) (McLaren and Callahan, 2021; Quast et al., 2013) or the manually curated GSR-DB database, which combines information from three different databases to eliminate inconsistencies in nomenclature and annotation, thereby increasing the resolution of the analysis (Molano et al., 2024). ASVs unclassified at the phylum level, as well as sequences identified as eukaryotic, archaeal, mitochondrial or chloroplast, were removed. After taxonomic filtering, 18 samples had fewer than 5,000 reads and were removed from further analyses, along with their corresponding M/T pairs. A total of 68 paired samples from each group (M or T) were ultimately included in the analysis, 34 from each participating institute.

The phyloseq package v 1.38 (McMurdie and Holmes, 2013) was used to generate phyloseq objects for further analyses. To eliminate spurious ASVs, sequences classified as the same species or unclassified at the species level were agglomerated. ASVs present in less than 1% of samples were removed.

#### 2.3.2 Bacterial composition, alpha and beta diversity

Alpha diversity indices were computed from phyloseq objects, and significant differences were assessed using the Wilcoxon signed-rank test. A neighbor-joining phylogenetic tree was constructed to obtain phylogenetic information, as previously described (Văcărean-Trandafir et al., 2023).

The Bray−Curtis distance matrix was computed from counts normalized through variance stabilizing transformation (VST), as implemented in the DESeq2 package (Love et al., 2014), while UniFrac distances were calculated from raw counts using the GUniFrac package v 1.6 (Chen et al., 2012). Differences in beta diversity between sample groups were assessed by permutational multivariate analysis of variance (PERMANOVA) on the Bray−Curtis distance matrix using the vegan package v 2.6-2 (Oksanen et al., 2014). Principal coordinate analysis (PCoA) of the Bray−Curtis and UniFrac distances was performed with the phyloseq package. Taxa that differed significantly between groups were identified from genus-agglomerated phyloseq objects using linear discriminant analysis effect size (LEfSe), implemented in the microbiomeMarker package v 1.0.2 (Cao et al., 2022), using an LDA cutoff of 4. All plots were generated using ggplot2 v 3.3.6 (Wickham, 2016).

## 3 Results

### 3.1 Participant characteristics

The initial cohort consisted of 400 adult individuals admitted for surgical oncology procedures divided into two groups of 200 patients from each oncological center. The subset of 86 colorectal cancer patients described herein was selected from this patient population. Paired samples (M - before surgery and T – 7 days after the end of antibiotic treatment) were obtained, resulting in 172 paired samples from colorectal cancer patients who underwent oncological colorectal surgery. Of the selected patients, 47 were male and 39 were female (aged 30 to 84 years, average age 64.52 years, median age 66 years). We further excluded 18 participants who had fewer than 5,000 reads after filtering steps, leaving 68 remaining subjects, of whom 36 were male and 32 were female. In the selected cohort, 27 patients had rectal tumors, 17 patients had tumors in the sigmoid colon, 2 had tumors in the hepatic flexure, 12 had tumors in the ascending colon, 3 had tumors in the transverse colon, 2 had tumors in the descending colon, 2 had tumors in the cecum and 3 had tumors generally classified as colon cancer. The demographic and medical data are available in the metadata table. According to institutional perioperative prophylaxis guidelines, all 36 patients from the IRO group received a single dose of a second-generation cephalosporin (cefuroxime) within 60 min preceding the incision, with up to two doses within 12 hours, depending on the length of the surgery, plus a single dose of 2 g metronidazole. The NIO cohort received various antibiotics, dosages and treatment durations, including first-generation cephalosporins (cefazolin, 7 patients) and third-generation cephalosporins (cefotaxime, 5 patients; ceftriaxone, 4 patients; and cefoperazone in combination with sulbactam, 18 patients).

### 3.2 Genomic DNA concentration

By combining both enzymatic and mechanical lysis over a longer incubation time (data not shown), we obtained higher DNA concentrations and purities, with average absorbance rates (A260/280) of 1.9 and 1.88 in the M and T group, respectively. The DNA concentration measured spectrophotometrically in the M samples varied from 172.7 ng/mL to 12.8 ng/mL (average 42.5 ng/mL), while that in the T samples ranged from 285.9 ng/mL to 12.3 ng/mL (average 30.7 ng/mL). Mechanical lysis by bead beating is strongly linked with bacterial diversity and is critical for successfully isolating the DNA of gram-positive bacteria for short-read sequencing technologies, such as the Illumina MiSeq platform (Lim et al., 2018; Wesolowska-Andersen et al., 2014; Wagner Mackenzie et al., 2015). By adding an additional step that consisted of lysozyme and lysostaphin-based enzymatic lysis in a bead-enzyme combination processing phase, we could effectively and accurately detect both gram-positive and gram-positive bacteria, resulting in a more realistic depiction of the gut microbiota. Consequently, enzymatic lysis may increase DNA yield and sensitivity of NGS approaches for gram-positive bacteria, and the addition of positive and negative controls during DNA isolation is strongly recommended for validation.

### 3.3 16S rRNA sequence read processing

A total of 26,107,340 reads were processed from the 172 sample pairs (average 151,785 reads per sample, median 81,382 reads per sample). After all filtering steps before taxonomic assignment, 12,731,516 sequences remained (average 74,040 filtered reads per sample, median 37,651 filtered reads), corresponding to 48.76% of the initial reads. The final amplicon sequence variant (ASV) table contained 11,077 unique bacterial sequences when taxonomic assignment was performed using the SILVA reference database and 11,009 unique bacterial sequences when taxonomic assignment was performed using the GSR reference database. After removing samples with fewer than 5,000 reads following all filtering steps, the ASV tables contained 10,255 and 10,180 unique bacterial sequences for the SILVA and GSR-classified sequences, respectively, with more than 28% of the unique sequences being singletons. Since multiple unique ASVs can be classified as belonging to the same species and many cannot be classified at the species level, leading to more complex datasets that are not necessarily biologically meaningful for various reasons (Reitmeier et al., 2021), we decided to consider overall taxonomic groups rather than individual sequence variants. Thus, the sequences were agglomerated at the species level, resulting in ASV tables containing 943 and 1001 unique taxa for the SILVA and GSR-classified sequences, respectively. It is important to note, however, that while agglomeration reduces complexity, it also results in a loss of fine-scale taxonomic resolution and may overlook important variations at the strain or subspecies level. Nevertheless, we considered this approach to be adequate for our dataset since our study focused on broader community patterns rather than individual sequence variants. After agglomeration, we eliminated ASVs that were present in fewer than 3% of the samples, resulting in ASV tables with 514 and 525 taxa for the SILVA- and GSR-classified sequences, respectively. Venn diagrams of taxa from the M (before antibiotic treatment) and T (7 days post antibiotic treatment) groups from both countries (RO – Romania and MD - Moldova) are available in **Supplementary Figure 1**.

### 3.4 Control samples

A total of 12 positive and 15 negative controls were processed. Among the negative controls, read numbers varied from 0 to 396 after all filtering steps, with an average of 143 reads per sample and low absolute counts per taxa, indicating minimal laboratory contamination. Therefore, all taxa identified in the negative control samples were not discarded from further analyses.

Among the positive controls, we performed three repetitions of four separate bacterial mock mixtures with defined community structures (CP1, CP2, CP27 and CP28). We found some variation from the expected relative abundances, with the highest bias observed for gram-negative *Klebsiella pneumoniae* (**Supplementary Figure 2**). For CP1 and CP2, the mean obtained relative abundances were 42.6% *Klebsiella* (SD = 2.8), 21.1% *Enterococcus* (SD = 1.2), 21% *Escherichia* (SD = 0.4) and 15% *Staphylococcus* (SD = 2.1; expected 25% for each genus). For CP27, the mean obtained relative abundances were 48.1% for *Escherichia* (SD = 1.3), 36.8% for *Staphylococcus* (SD = 1.2) and 15.1% for *Enterococcus* (SD = 0.2; expected 50% for *Escherichia*, 25% for *Staphylococcus* and 25% for *Enterococcus*). Finally, for CP28, the mean obtained relative abundances were 50.3% for *Serratia* (SD = 1.4), 24.6% for *Staphylococcus* (SD = 1.1), 18.4% for *Enterococcus* (SD = 1.2) and 6.7% for *Escherichia* (SD = 0.2; expected 45% for *Serratia*, 25% for *Staphylococcus*, 25% for *Enterococcus* and 5% for *Escherichia*). This finding suggested that the combined DNA extraction protocol was suitable for extracting both gram-positive and gram-negative bacteria, albeit with slight bias toward gram-negative strains.

### 3.5 Alpha diversity

**Figure 1:**
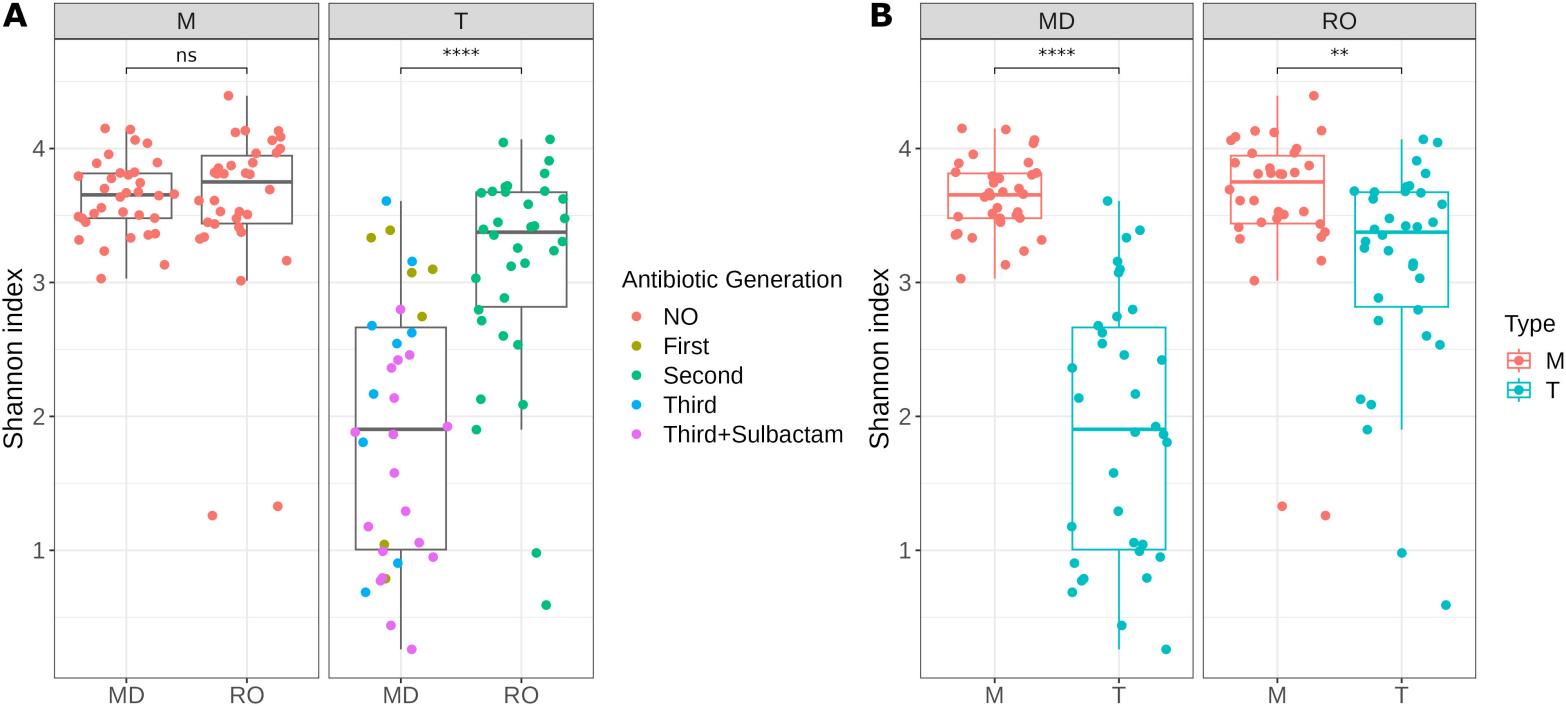
Shannon Index as a measure of alpha diversity for samples before (M) and 7-days post antibiotic treatment (T) from IRO (RO) and NIO (MD); **(A)** Sample grouping based on sample type; Mann-Whitney test used for statistical significance; **(B)** Sample grouping based on sample origin; Wilcoxon signed-rank test used for statistical significance; ** p<0.01, **** p<0.0001; ns – not significant; NO – no antibiotic treatment; First – first-generation cephalosporin; Second – second-generation cephalosporin; Third - third-generation cephalosporin; Third + Sulbactam - third-generation cephalosporin in combination with Sulbactam.

We found no significant difference in terms of alpha diversity between country-specific M samples (before antibiotic administration; **Figure 1A**), suggesting that the microbial composition was similar between the two study groups before antibiotic treatment. However, we observed that alpha diversity was significantly greater in T samples from IRO than in T samples from NIO, which received a more diverse set of antibiotic treatment types (Mann-Whitney test, *P* = 2.34 × 10^-5^). Additionally, compared with pretreatment, both institutional antibiotic prophylaxis practices induced a significant decrease in alpha diversity (**Figure 1B**), with the effect being more pronounced for NIO patients.

### 3.6 Bacterial composition

Comparing the most prevalent taxa at the class level between samples led to a clear separation of groups in terms of the type of antibiotic administered (**Supplementary Figure 3**). *Bacilli, Bacteroidia, Clostridia*, and *Gammaproteobacteria* were the dominant classes across all the samples, with noticeable changes in relative abundances after antibiotic treatment. Notably, in patients from the IRO group receiving the second-generation cephalosporin cefuroxime, the average bacterial composition in terms of relative abundance at the class level was shifted from 6.3% to 11.7% for *Bacilli*, from 42.5% to 27.8% for *Clostridia*, and from 7.9% to 16.6% for *Gammaproteobacteria* before and 7 days after antibiotic treatment. The class *Bacteroidia* exhibited approximately the same relative abundance (34.4% and 35.2%, respectively; **Supplementary Figure 3**). In the case of NIO patients receiving first-generation antibiotic treatment, a similar trend was observed, with average class relative abundance values shifting from 3.1% to 20.5% for *Bacilli*, from 41.6% to 14.7% for *Clostridia*, from 4.6% to 21.9% for *Gammaproteobacteria*, and comparable *Bacteroidia* levels before and 7 days after antibiotic treatment (40.3% and 37.2%, respectively).

In the case of NIO patients receiving third-generation antibiotic treatment alone or in combination with sulbactam, the average class-level relative abundance values shifted as follows: *Bacilli* increased from 3.1% to 38.1% with third-generation cephalosporins alone and to 63.2% with the combination treatment; *Clostridia* decreased from 41.6% to 11.8% with third-generation cephalosporins alone and to 6.5% with the combination treatment; *Gammaproteobacteria* increased from 4.6% to 13.1% with third-generation cephalosporins alone and to 10.7% with the combination treatment; *Bacteroidia* decreased from 40.3% to 25.5% with third-generation cephalosporins alone and to 6.2% with the combination treatment, before and 7 days post antibiotic treatment.

**Figure 2:**
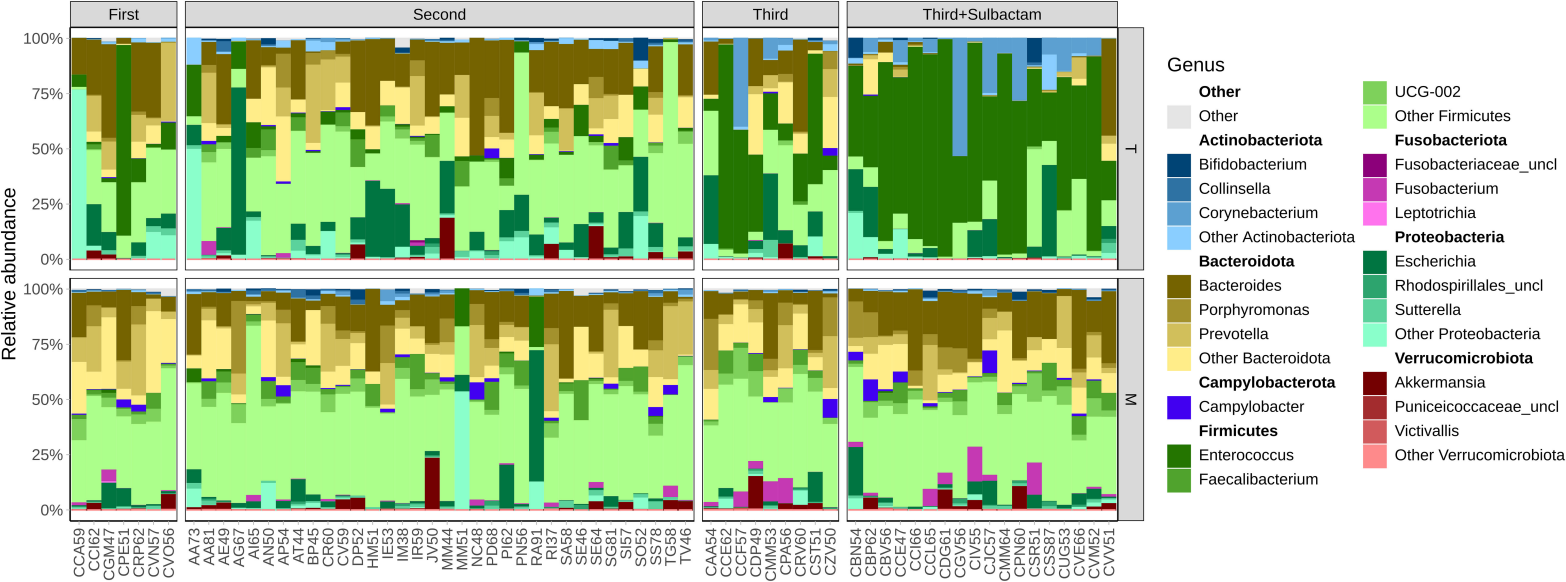
Bacterial community structure at genus level per each group, in terms of relative abundances before (M) and 7-days post antibiotic treatment (T) from IRO and NIO; First – first-generation cephalosporin; Second – second-generation cephalosporin; Third, third-generation cephalosporin; Third + Sulbactam, third-generation cephalosporin in combination with Sulbactam; corresponding phyla highlighted in bold above each genus group.

At the genus level, it became evident that the main increase in the abundance of the class *Bacilli* in NIO patients receiving a combination of cefoperazone and sulbactam was due to an overall increase in *Enterococcus* abundance (**Figure 2**). This patient group also had a greater abundance of the genus *Corynebacterium*, while bacteria in the phylum *Bacteroidota* were nearly absent.

**Figure 3:**
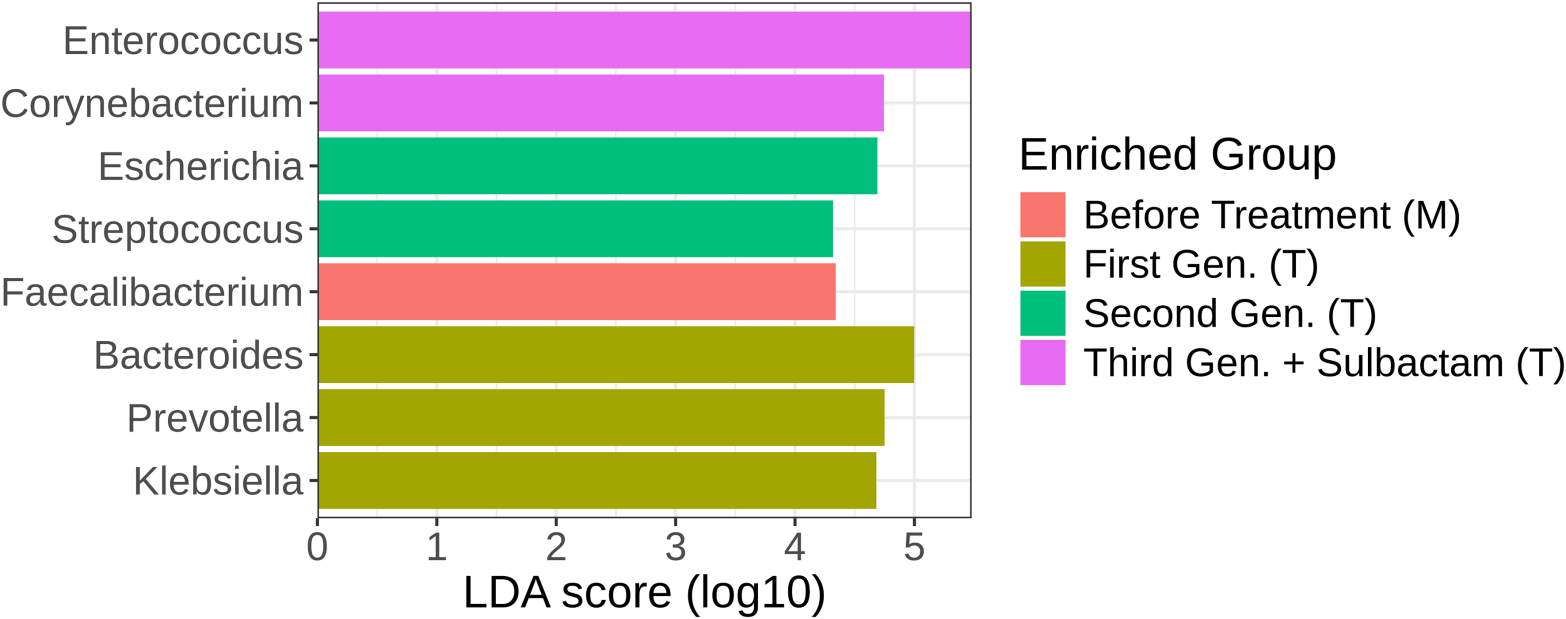
LDA scores of genus-level taxa obtained using LEfSe analysis. The colors represent the sample types: red – Before antibiotic treatment (M); olive – 7 days post treatment with first-generation cephalosporins (NIO); green – 7 days post treatment with second-generation cephalosporins (IRO); and purple – 7 days post treatment with third-generation cephalosporins + sulbactam (NIO).

Differential abundance analysis confirmed that samples from patients receiving a combination of third-generation cephalosporins and sulbactam were enriched in *Enterococcus* and *Corynebacterium.* Additionally, samples before antibiotic treatment, regardless of the patients’ country origin, were significantly more abundant in *Faecalibacterium* than any of the treatment groups (**Figure 3**). This genus, particularly the species *Faecalibacterium prausnitzii*, is a key component of the commensal gut microbiota and plays an important role in maintaining gut health (Parsaei et al., 2021). This finding suggests that this genus was affected regardless of the administered antibiotic and did not regain pre-treatment abundance levels 7 days post antibiotic treatment. Samples from patients receiving second-generation cephalosporin cefuroxime were enriched in the genera *Escherichia* and *Streptococcus*, indicating that these opportunistic infectious agents are more likely to repopulate the gut faster than in the other evaluated antibiotic treatments. Similarly, samples from patients receiving first-generation cephalosporins were enriched in *Klebsiella.* Furthermore, these samples were also enriched in intestinal commensals from the genera *Prevotella* and *Bacteroides.* This suggests that first-generation cephalosporins are less effective at eliminating these gut commensals than are the other tested cephalosporins, as others have also noted (Brook, 2017). In the absence of competition from susceptible bacteria, *Prevotella* and *Bacteroides* appear to proliferate more abundantly in the gut of patients receiving this type of treatment. While these two genera can be beneficial to the gut, the recent discovery of the involvement of certain *Prevotella* species in the malignant transformation of colorectal adenomas (Lo et al., 2022) indicates that caution should be taken against the use of antibiotics that favor *Prevotella* proliferation. Our data highlights significant shifts in microbial communities depending on the type of antibiotic administered, with specific genera being enriched in particular groups. This underscores the profound impact of antibiotic treatments on the intestinal microbiota and the importance of considering these effects when managing gut health and developing therapeutic strategies.

Thus, the degree of compositional changes varies greatly depending on the type of antibiotic used, with third-generation cephalosporins (alone or combined with sulbactam) having more pronounced effects on the microbial composition. These observations suggest that the choice of antibiotic can significantly influence the gut microbiota, potentially leading to dysbiosis or shifts in microbial community structure.

### 3.7 Beta diversity

**Figure 4:**
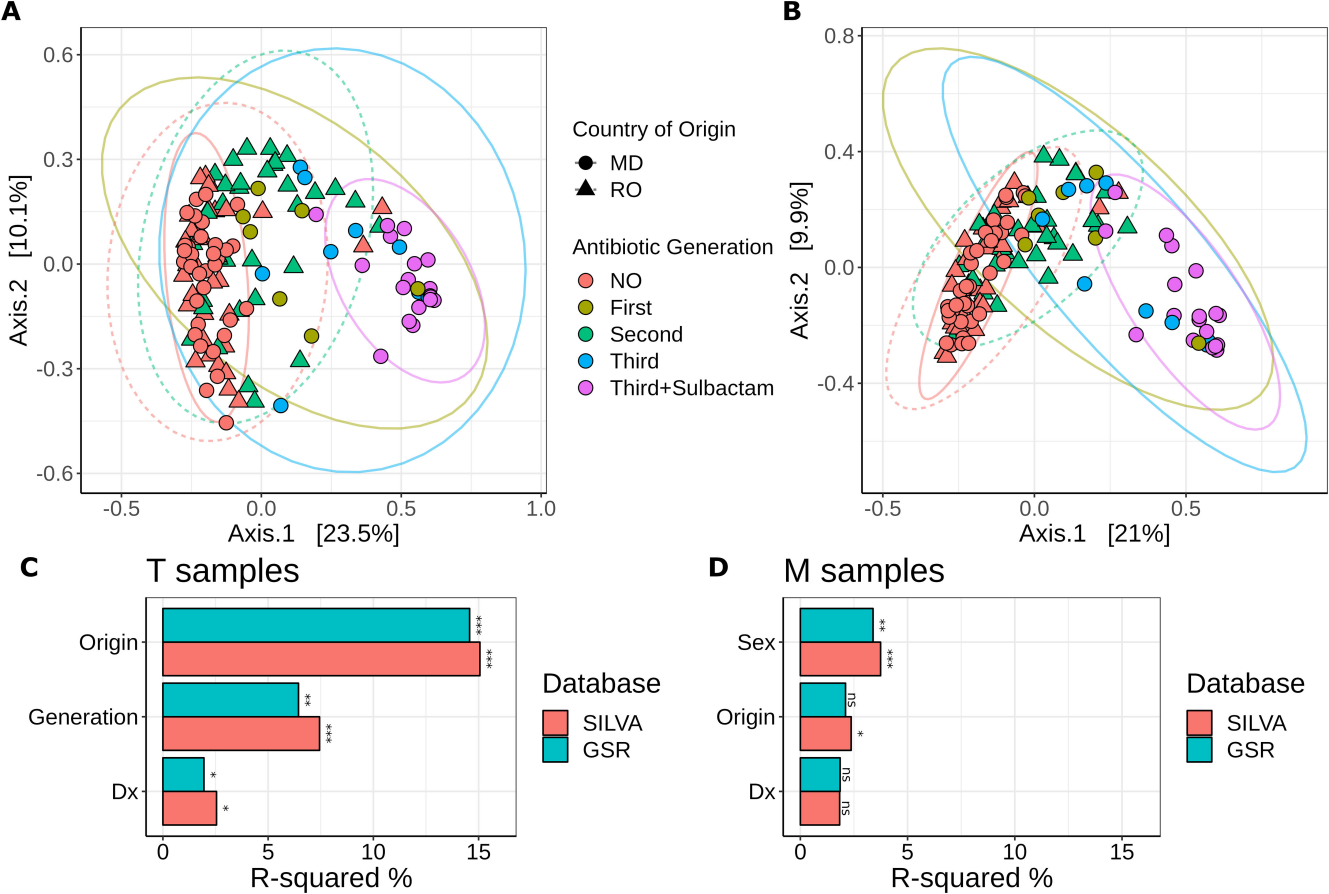
**(A, B)** PCoA plots of Bray−Curtis dissimilarity from relative abundance data illustrating distances between communities in individual samples following taxonomic assignment using **(A)** SILVA and **(B)** GSR as reference database. Point shapes indicate country of origin: circles – samples from NIO (MD); triangles – samples from IRO (RO). Colors represent sample types: red – No antibiotic treatment (NIO and IRO); olive – 7 days post treatment with first-generation cephalosporins (NIO); green – 7 days post treatment with second-generation cephalosporins (IRO); blue – 7 days post treatment with third-generation cephalosporins alone (NIO); purple – 7 days post treatment with third-generation cephalosporins in combination with sulbactam (NIO); Ellipses are drawn at 95% confidence level. The percentage of variation explained by the first two dimensions is indicated on respective axes; **(C, D)** PERMANOVA based on Bray−Curtis dissimilarity from relative abundance data between T and M samples. Origin – country of origin (MD or RO); Generation – Antibiotic generation; Dx – cancer type. *p<0.05, **p<0.01, ***p<0.001; ns, not significant.

PCoA analysis using the Bray−Curtis dissimilarity matrix upon taxonomic assignment using both the SILVA and GSR reference databases showed a clear separation of samples based on the type of antibiotic administered, with third-generation cephalosporins alone or in combination with sulbactam showing distinct clusters separate from other treatments or no treatment groups (**Figure 4**). There was some overlap between samples before antibiotic treatment from both countries and 7 days after first-generation and second-generation antibiotic treatment. Notably, taxonomic assignment using SILVA led to slightly better segregation of groups, with more variation explained by the PCoA axes. The differences in clustering between results using the same metrics but different databases highlight the importance of the choice of reference database in microbiome studies. Overall, these results suggest that the type of antibiotic administered strongly influences the gut microbial composition, with potential implications for gut health and dysbiosis.

PERMANOVA based on Bray−Curtis distances following taxonomic assignment with SILVA showed that the greatest determinant of intestinal microbiota structure in samples 7 days post antibiotic treatment (T) was country of origin (R^2^ = 15%, *p* = 0.001), followed by treatment type (R^2^ = 7.4%, *p* = 0.001). To a lesser extent, cancer type (Rectal versus Colon - R^2^ = 2.5%, *p* = 0.004) also contributed to the variability (**Figure 4C**). However, the duration of treatment was not found to significantly contribute to the observed compositional variation between samples. Similar results were obtained for PERMANOVA based on Bray−Curtis distances following taxonomic assignment with GSR. Differences in post-operative diet and healthcare practices between countries could explain further variability, but these factors were not taken into account in this study. In contrast, PERMANOVA on samples before antibiotic treatment (M) following taxonomic assignment with SILVA showed that country of origin contributed to a far lesser extent to the intersample compositional variability (R^2^ = 2.3%, *p* = 0.025), suggesting that the bacterial composition of the samples before antibiotic treatment was similar in both countries. We observed that patient sex accounted for some of the variability in the samples before antibiotic treatment (R^2^ = 3.7%, *p* = 0.001) (**Figure 4D**). Cancer type was not significantly linked to compositional variability (R^2^ = 1.83%, *p* = 0.118). Similar results were obtained following taxonomic assignment with GSR, albeit the R^2^ values were usually lower (**Figures 4C, D**). Overall, these results suggest that both intrinsic factors (such as patient sex and cancer type) and extrinsic factors (such as country of origin and antibiotic treatment) play important roles in shaping the gut microbiota. Understanding these influences can help in developing more personalized and effective therapeutic strategies to manage gut health and mitigate dysbiosis following surgical interventions. Our results also highlight that both SILVA and GSR are suitable as reference databases and lead to comparable results.

## 4 Discussion

Understanding the physiology of the intestinal microbiota and its disruption under antibiotic stress is a critical milestone for those who work as doctors, healthcare personnel, and patients. Antibiotic-resistant bacteria have become central to many hospital-acquired infections, with careless antibiotic usage serving as the trigger for this harmful cycle. Our research seeks to elucidate the impact of antibiotics on the gut microbiota by identifying the antibiotics with the most profound effects on the microbiome, determining the duration of these effects, and distinguishing bacterial species that are predisposed to such impacts.

The absence of a consistent approach makes quantifying the efficacy and risks associated with antibiotic abuse difficult, particularly since not only the type of antibiotic but also the dose and duration are uncontrolled. If the integrity of the microbiome is not considered when selecting a treatment, the repercussions could be disastrous for both the patient and the hospital, as complications related to antibiotic misuse will have a major financial impact. Given these factors, it is imperative to prioritize research aimed at understanding the effects of antibiotics on the microbiome and enhancing the efficiency and gut-friendliness of antibiotic treatments.

The use of antibiotics significantly alters the ecology of the intestinal microbiota and permanently impacts both developing and adult microbiota (Zimmermann and Curtis, 2019; Dierikx et al., 2020; Andremont et al., 2021). The adoption of next-generation sequencing has enabled researchers to examine in great detail how these medications affect commensal communities while managing infections (Yu et al., 2017; Kharofa et al., 2023). The intestinal microbial community plays a pivotal role in shaping human well-being, and various health conditions have been associated with microbiome dysbiosis (Belizario and Faintuch, 2018; Das and Nair, 2019; Dupont et al., 2020; Sharma et al., 2021). Achieving a complete understanding of the microbiome’s true functionality and its varied characteristics will require the implementation of multiple cohort studies and synthesize their results in large-scale meta-analyses. As in any other scientific field, inconsistent methodological procedures pose a significant challenge to these comparative efforts. While every stage in the sequencing-based examination of the microbiome is likely important, it is broadly acknowledged that the DNA extraction procedure is pivotal for shaping the variation and accuracy of detecting intricate bacterial populations (Kazantseva et al., 2021; Videnska et al., 2019; Kool et al., 2023; Liu et al., 2024; Hong et al., 2024). Given that the gut microbiota encompasses both gram-positive and gram-negative bacteria with widely varying abundance levels, it is crucial to select an unbiased genomic DNA extraction approach capable of handling these challenges and establishing consistent reproducibility within and among diverse research laboratories. Research findings have indicated that mechanical lysis via bead beating accompanied by a cocktail of lytic enzymes is positively correlated with bacterial diversity and is an indispensable step for effectively isolating the DNA of gram-positive bacteria.

According to WHO surveillance data from 2021, only 33% of countries have guidelines and practices to optimize the use of antimicrobials that are being implemented nationwide in most health facilities, with surveillance results used to update treatment guidelines and essential medicines lists. Prominent projects in Europe, the United States, China, and Canada followed by additional ones from Africa and Australia target these objectives using advanced high-throughput omics methods. These projects aim to comprehensively characterize our microbial communities, which are intricate and diverse and consist of numerous species that vary significantly between individuals and exert various impacts on our biology. To efficiently advance toward this ambitious goal, it is critical that the data generated in each individual project are optimally comparable across all current and future projects. Although there are several ASPs in Western Europe (in France, Germany, Ireland, the Netherlands, Spain and the UK), antimicrobial resistance poses a greater challenge to LMICs from Eastern Europe and around the globe since they also struggle with resource availability issues. A review published in 2020 aimed to assess the efficacy of SAP in reducing SSI in low- and middle-income countries and concluded that, despite healthcare providers undoubtedly confronting environmental obstacles amplified by limited resources, enhancing practices can yield positive results even in resource-constrained settings (Pierce et al., 2020).

The objective of this study was to characterize the intestinal microbiota of a cohort of surgical CRC patients from two distinct populations. We aimed to determine whether the overall microbial community composition differed between these groups and to distinguish bacterial taxa across the groups upon separate antibiotic prophylaxis. The antibiotic regimens administered preoperatively differed between the two populations; nevertheless, comparing the effects of antibiotics on the microbiota was straightforward due to the similarity in the gut microbiota among the examined populations. First, a large cohort of patients with similar diets and environmental exposures was employed for the study. The Republic of Moldova and the Moldavia Region of Romania are sibling communities with longstanding coexistence and common dietary practices. The distance between two hospital units is 173 km, which is less than any other similar units in other countries, which is advantageous for comparing two different populations with resembling surroundings. While IRO has very rigorous guidelines on prophylactic antibiotic therapy (single dose, single product, restricted period), at NIO each surgical team has significant flexibility, enabling them to choose the antibiotic and its duration at their discretion. The wider the antibiotic’s spectrum and the longer it is used, the greater the influence on the human microbiome. Given this variation, we can assess our hypothesis without compromising standard practice or patient safety. Although each person possesses a unique combination of bacterial populations, we anticipated the occurrence of a common pattern among patients before antibiotic treatment. Consequently, the primary factor contributing to significant variations in individual microbiomes should originate from varying antibiotic regimens. As leading oncological centers in these respective areas, we consolidated our oncological care and had access to similar patient populations. Our statistical findings revealed substantial differences in the prophylactic use of antibiotics among nonseptic patients.

Several reviews of the literature advocate that the use of third-generation cephalosporins, such as ceftriaxone, cefotaxime or cefoperazone, has increased resistance among different classes of bacteria, thus disrupting the intestinal microecology and resulting in the overproliferation of certain species (Konstantinidis et al., 2020; Huang et al., 2023; Pilmis et al., 2020). In contrast, other studies support the use of a single dose of cefuroxime (in combination with metronidazole) for antibiotic prophylaxis, as it does not have a major effect on the gut microbiota (Sastry et al., 2021; Kamal et al., 2019).

Another major advantage of cross-border collaboration is the ability to effectively manage and correlate the intestinal microbiota of two different yet comparable populations. After examining the effects of various antibiotic regimens on the digestive tract microbiota, the next step would be to track long-term changes in the microbiome due to clinical factors, cancer status, or age. With these questions addressed, doctors will be able to improve their antibiotic strategies and learn how to select antibiotics that are best suited to the patient’s microbiome environment. Considering this, our research effort will present modified and standardized antibiotic regimens to assist clinicians in choosing the most suitable strategy.

The study presented herein highlights significant findings regarding the effects of different antibiotic treatments on the diversity and composition of the gut microbiota. Key findings include no significant difference in alpha diversity between country-specific samples before antibiotic administration, indicating similar microbial compositions. Post-treatment, there was a significant decrease in alpha diversity, which was more pronounced in NIO patients than in IRO patients, where samples showed greater variability due to varied antibiotic regimens. Antibiotic treatments led to notable changes in the relative abundance of dominant microbial classes. For example, second-generation cephalosporin treatment (IRO) resulted in an increase in *Bacilli* and *Gammaproteobacteria* and a decrease in *Clostridia*. First-generation cephalosporin treatment (NIO) had a similar effect, with significant increases in *Bacilli* and *Gammaproteobacteria* and a decrease in *Clostridia*. Third-generation cephalosporin treatment (NIO) led to a dramatic increase in the abundance of *Bacilli*, especially when combined with sulbactam, and a reduction in the abundance of *Clostridia* and *Bacteroidia*. PCoA revealed distinct microbial community clusters based on the type of antibiotic used, with third-generation cephalosporins causing the most pronounced changes. PERMANOVA identified country of origin and treatment type as major determinants of microbiome structure post-treatment, with patient sex and cancer type also contributing to some variability. Differential abundance analysis revealed that bacteria from the genus *Faecalibacterium* was significantly reduced across all post-treatment groups. Additionally, specific antibiotics enriched opportunistic pathogens such as *Escherichia* and *Streptococcus* (second-generation cephalosporins), *Enterococcus* and *Corynebacterium* (third-generation cephalosporins with sulbactam), and *Prevotella* and *Bacteroides* (first-generation cephalosporins).

Our intention was to use these data to inform hospital administrations, governmental authorities, and local communities about the necessity of enforcing stringent controls on antibiotic utilization and revising the practices of antibiotic prophylaxis. Implementing proper antibiotic usage will yield direct cost reductions (through the decreased use of expensive antibiotics) and additional benefits by reducing complications and containing multiresistant pathogens within the hospital environment.

We employed these findings to create and facilitate interactive learning sessions involving various healthcare professionals at the two medical centers. The aim was to share the research evidence and encourage adherence to both the WHO and unanimous national guidelines. In collaboration with their antimicrobial stewardship teams, both hospitals are formulating evidence-based local SAP policies tailored to various surgical subspecialties. These local policies are being disseminated to clinical staff through presentations and posters, as well as through continuous training integrated into stewardship programs. We anticipate that this effort will be valuable to other nations seeking to enhance antimicrobial usage, reduce elevated rates of antimicrobial resistance (AMR), and implement effective ASPs to optimize surgical antibiotic prophylaxis.

## 5 Strengths, limitations and future directions

Other research organizations may plan to adopt and expand this research model for broader regions, recognizing the need for a national shift. Overall, most patients are affected by inadequate antibiotic protocols, as they experience pathologic distress due to dysbiosis. Our findings highlight the advantages of microbiome-friendly antibiotic regimens for patients, as they can improve their hospital experience and lower the risk of *Clostridioides difficile* or other bacterial infections, which are known to be influenced by antibiotic exposure (Miller et al., 2023; McDonald et al., 2018). However, it is important to note that we did not specifically measure reductions in *C. difficile* infection rates in this study, although this could be the subject of a subsequent analysis.

The strengths of our research include the use of state-of-the-art next-generation sequencing and bioinformatic tools/analyses, the use of a cross-border homogenous study population, and the use of a prospective study design, thus reducing the risk of biases associated with retrospective studies. However, it is important to point out that while partial 16S rRNA gene sequencing provides valuable information about the composition of bacterial communities, it primarily identifies taxa at the genus level, which may obscure important species-level distinctions and functional attributes of the microbiota. More comprehensive methods, such metagenomics, could yield deeper insights into microbiota alterations by enabling the analysis of the entire microbial community, including viruses, fungi, and archaea, and by providing functional data that reveals the metabolic pathways and interactions within the microbiome. Therefore, while our study utilized the V3-V4 region of the 16S rRNA gene to identify relevant changes, we acknowledge that future research incorporating metagenomic techniques could significantly enhance our comprehension of the complex dynamics within the gut microbiota.

The primary limitation of this study is the relatively small sample size, along with the short duration of follow-up of the intestinal microbiota. The limited sample size may also affect the outcomes of both univariate and multivariate analyses, which in turn impacts the generalizability of our findings. Of note, the lack of data regarding taxonomic changes at later postintervention timepoints urges for future investigations to determine if the identified differentially abundant taxa are outcompeted by commensal bacteria in time. It might have been interesting to examine the dynamics of the recovery of various taxa and the underlying reasons for this process. Additionally, our study did not take into account differences in surgical techniques, hospital hygiene practices or patient diets between the two countries nor did we consider the local incidence in multi-resistant infection rates. Future studies could benefit from incorporating these variables to further refine the analysis and control for additional confounding factors. Despite these limitations, we provide intriguing and original results which could be further explored through more extensive research.

Continuous process improvement approaches provide considerable clinical benefits in treating major postoperative comorbidities in oncological patients. These investigations have revealed the next areas for improvement. Future research should focus on cefuroxime-metronidazole antibiotic prophylaxis for individuals who are allergic to these drugs or to include dietary habits, probiotic intake, BMI data and diabetes status. Another important step will be to evaluate the plasticity of the colonic microbiota after antibiotic intervention, assessing its capacity and extent of recovery within a month. Additionally, considering the beta-lactamase activity within fecal samples as an indicator of the intestinal microbiota’s exposure to beta-lactam antibiotics could provide valuable insights.

Understanding the modifications occurring within the gut microbiota of colorectal cancer surgery patients is highly important, yet this topic has not yet been fully explored. Additional large-scale prospective research studies, particularly those associated with mechanical bowel preparation and the use of nonabsorbable oral antibiotics, should be considered. These studies should also take into account other contributing factors to obtain results that can be verified and applied universally.

## 6 Conclusions

The current investigation established that the application of a local protocol, adherence to treatment, frequent assessments of perioperative antibiotic prophylaxis, and effective teamwork among healthcare practitioners have effectively enhanced the prudent use of cefuroxime as a surgical preventative measure.

The DNA extraction technique was emphasized as a key element in shaping the overall bacterial makeup in a sample. Commercial isolation kits on the market employ various approaches for breaking down bacterial cells, including enzymatic, chemical, or mechanical methods. In most cases, enzymatic and mechanical disruption are advised to be used in conjunction to improve the lysis of gram-positive bacteria. Additionally, our study highlights the need for careful consideration of reference databases in microbiome research, as these can influence the interpretation of microbial community structures to some extent.

Modifications caused by broad-spectrum antibiotics can produce measurable effects on the human intestinal bacterial community, leading to immediate impacts on health status. In this research, our aim was to provide tangible evidence of antibiotic-driven alterations and advocate for a novel, more constrained approach to antibiotic usage, both as a cost-saving measure and to mitigate the emergence of multiresistant pathogens.

Antimicrobial resistance is an acknowledged and increasing concern worldwide, with LMICs bearing an unequal burden due to their scarce resources. Given the current trajectory, governments and healthcare institutions must prioritize decreasing resistance to antibiotics. Antimicrobial stewardship programs are excellent tools that require an organized implementation of research-supported measures aimed at optimizing antibiotic use and delaying the onset of antibiotic resistance.

## Data Availability

The datasets presented in this study are availabe on Zenodo and can be accessed at https://doi.org/10.5281/zenodo.11440440. The scripts used for the generation of presented results and figures are available upon request from corresponding authors.
