# Supplementary material for: Impact of antibiotic prophylaxis on gut microbiota in colorectal surgery: insights from an Eastern European stewardship study
Source: Front Cell Infect Microbiol. 2025 Jan 13;14:1468645. doi: 10.3389/fcimb.2024.1468645 (PMC11770057; doi:10.3389/fcimb.2024.1468645)
Supplement: Supplementary file 2 [file DataSheet2.docx]

***Supplementary Material***

**Impact of Antibiotic Prophylaxis on Gut Microbiota in Colorectal Surgery: Insights from an Eastern European Stewardship Study**

**Irina Cezara Văcărean-Trandafir^1,*,§^, Roxana-Maria Amărandi^1,*,§^, Iuliu Cristian Ivanov^1^, Loredana Mihaiela Dragoș^1^, Mihaela Mențel^1^, Ştefan Iacob^2,3^, Ana-Maria Muşină^2,3^, Elena-Roxana Bărgăoanu^2^, Cristian Ene Roată^2,3^, Ștefan Morărașu^2,3^, Valeri Țuțuianu^4^, Marcel Ciobanu^5^, Mihail-Gabriel Dimofte^2,3^**

^1^TRANSCEND Research Centre, Regional Institute of Oncology, 2-4 General Henri Mathias Berthelot Street, 700483 Iasi, Romania

^2^Second Surgical Oncology Department, Regional Institute of Oncology, 2-4 General Henri Mathias Berthelot Street, 700483 Iași, Romania

^3^Surgery Department, “Grigore T. Popa” University of Medicine and Pharmacy, 16 University Street, 700115 Iași, Romania

^4^Scientific Laboratory of Cancer Biology, Institute of Oncology, 30 Nicolae Testemitanu Street, MD-2025, Chișinău, Republic of Moldova

^5^Surgical Oncology Department, Proctology, Institute of Oncology, 30 Nicolae Testemitanu Street, MD-2025, Chișinău, Republic of Moldova


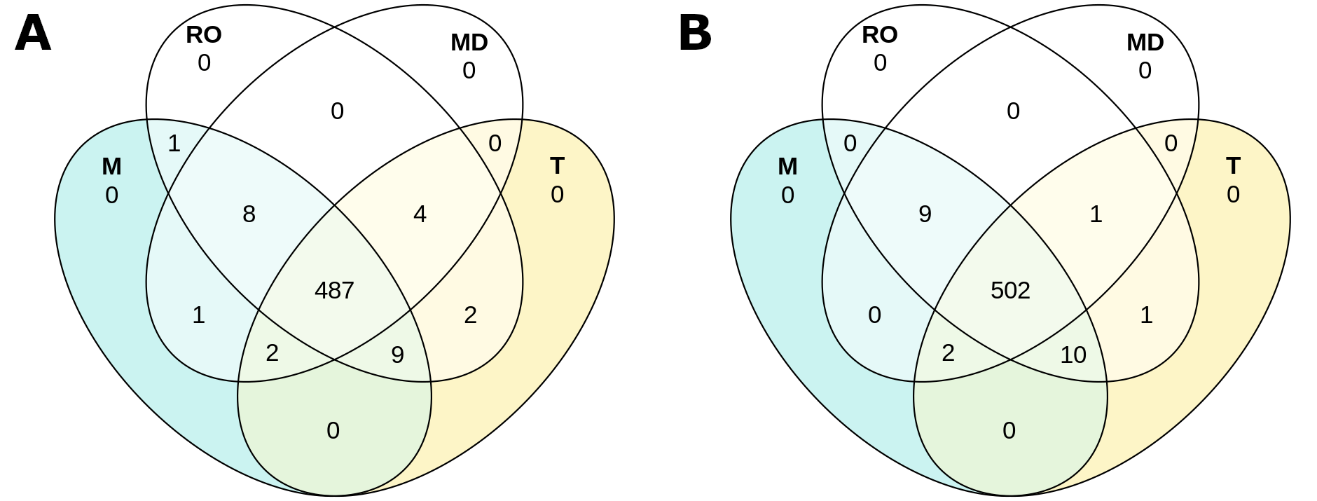


**Supplementary Figure 1. Number of taxa from groups following taxonomic assignment, agglomeration and prevalence filtering.** Groups are either M (before antibiotic treatment) or T (7 days post-antibiotic treatment) from both countries (RO or MD). (A) SILVA reference database used for taxonomic assignment and (B) GSR SILVA reference database used for taxonomic assignment


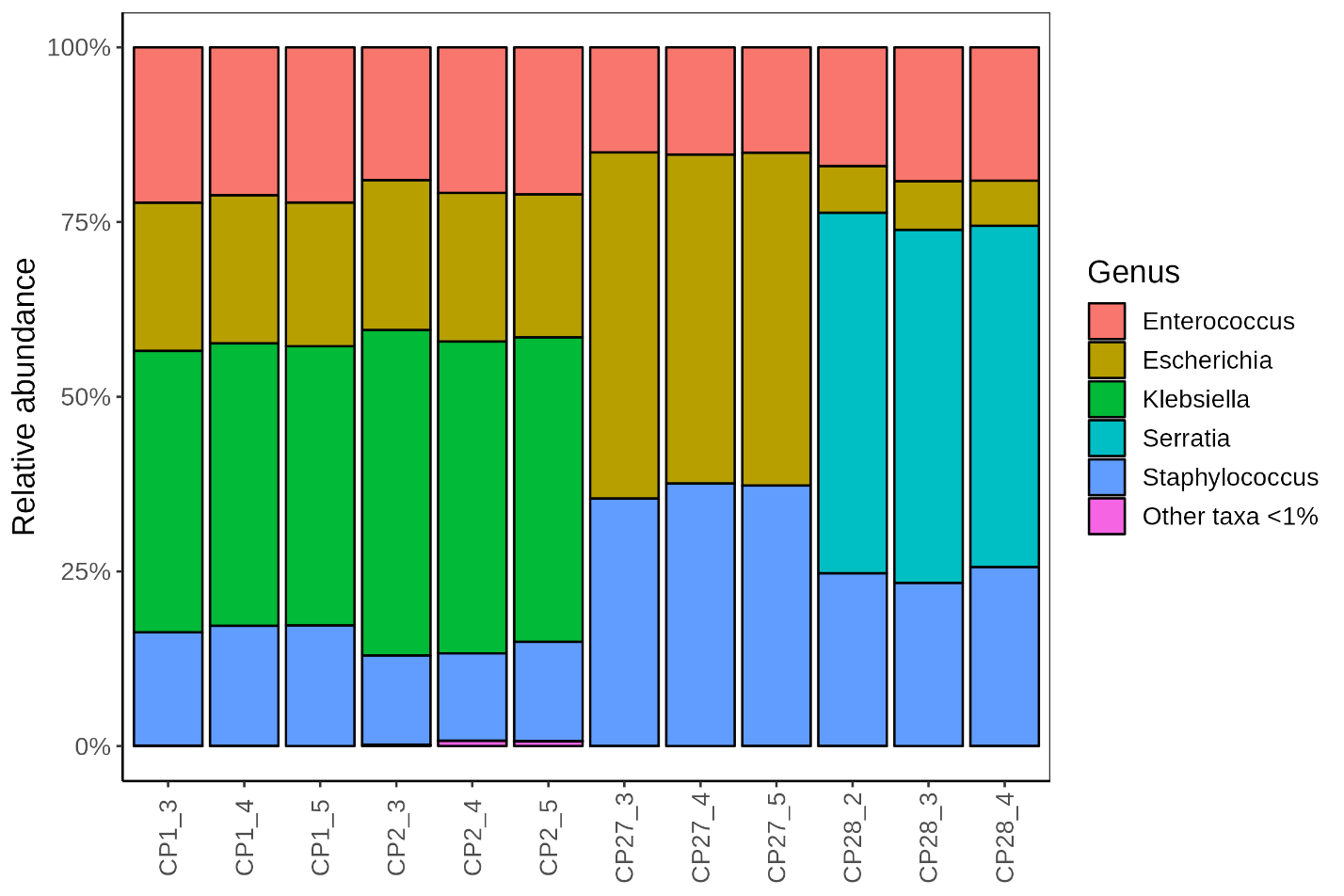


**Supplementary Figure 2. Community structure in positive controls.** Mock bacterial community structure at genus level per each positive control, in terms of relative abundances.


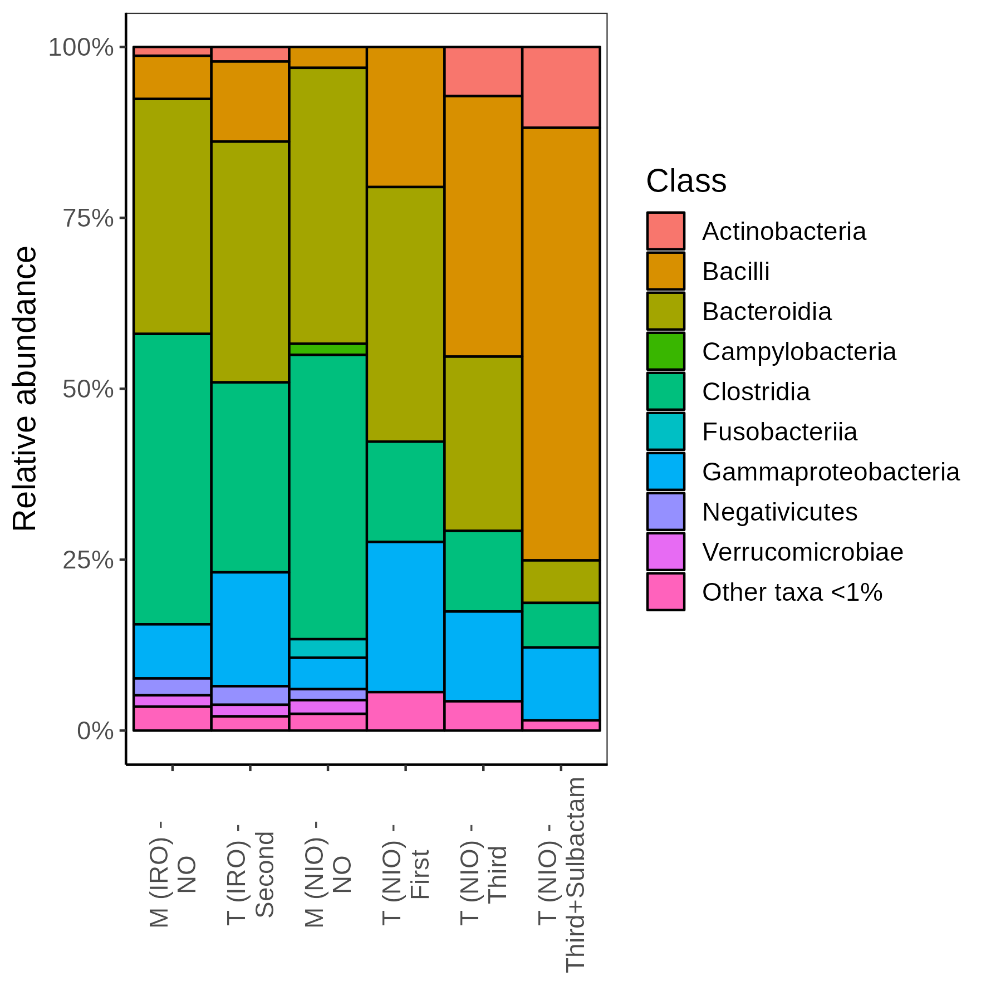


**Supplementary Figure 3. Average bacterial community structure.** Bacterial community structure merged at class level per each group, in terms of relative abundances before (M) and 7-days post antibiotic treatment (T) from IRO and NIO; NO – no antibiotic treatment; First – first-generation cephalosporin; Second – second-generation cephalosporin; Third - third-generation cephalosporin; Third+Sulbactam - third-generation cephalosporin in combination with Sulbactam; Classes with lower than 1% relative abundance are grouped together under ‘Other taxa <1%
